# The Association Between Telemedicine Use and Changes in Health Care Usage and Outcomes in Patients With Congestive Heart Failure: Retrospective Cohort Study

**DOI:** 10.2196/36442

**Published:** 2022-08-04

**Authors:** Cherry Chu, Vess Stamenova, Jiming Fang, Ahmad Shakeri, Mina Tadrous, R Sacha Bhatia

**Affiliations:** 1 Women's College Hospital Institute for Health System Solutions and Virtual Care Toronto, ON Canada; 2 Institute for Clinical Evaluative Sciences Toronto, ON Canada; 3 Leslie Dan Faculty of Pharmacy University of Toronto Toronto, ON Canada; 4 Ontario Health Toronto, ON Canada; 5 University Health Network Toronto, ON Canada

**Keywords:** telemedicine, telehealth, eHealth, digital health, population, outcomes, health service, health system, utilization, congestive heart failure, cardiology, health outcome, clinical outcome, patient outcome, heart, cardiac, cardiology, ambulatory, COVID-19

## Abstract

**Background:**

Telemedicine use has become widespread owing to the COVID-19 pandemic, but its impact on patient outcomes remains unclear.

**Objective:**

We sought to investigate the effect of telemedicine use on changes in health care usage and clinical outcomes in patients diagnosed with congestive heart failure (CHF).

**Methods:**

We conducted a population-based retrospective cohort study using administrative data in Ontario, Canada. Patients were included if they had at least one ambulatory visit between March 14 and September 30, 2020, and a heart failure diagnosis any time prior to March 14, 2020. Telemedicine users were propensity score–matched with unexposed users based on several baseline characteristics. Monthly use of various health care services was compared between the 2 groups during 12 months before to 3 months after their index in-person or telemedicine ambulatory visit after March 14, 2020, using generalized estimating equations.

**Results:**

A total of 11,131 pairs of telemedicine and unexposed patients were identified after matching (49% male; mean age 78.9, SD 12.0 years). All patients showed significant reductions in health service usage from pre- to postindex visit. There was a greater decline across time in the unexposed group than in the telemedicine group for CHF admissions (ratio of slopes for high- vs low-frequency users 1.02, 95% CI 1.02-1.03), cardiovascular admissions (1.03, 95% CI 1.02-1.04), any-cause admissions (1.03, 95% CI 1.02-1.04), any-cause ED visits (1.03, 95% CI 1.03-1.04), visits with any cardiologist (1.01, 95% CI 1.01-1.02), laboratory tests (1.02, 95% CI 1.02-1.03), diagnostic tests (1.04, 95% CI 1.03-1.05), and new prescriptions (1.02, 95% CI 1.01-1.03). However, the decline in primary care visit rates was steeper among telemedicine patients than among unexposed patients (ratio of slopes 0.99, 95% CI 0.99-1.00).

**Conclusions:**

Overall health care usage over time appeared higher among telemedicine users than among low-frequency users or nonusers, suggesting that telemedicine was used by patients with the greatest need or that it allowed patients to have better access or continuity of care among those who received it.

## Introduction

The COVID-19 pandemic has significantly increased the adoption of telemedicine globally, with governments reducing regulatory restrictions on telemedicine platforms and funding telemedicine visits with new billing codes [1]. Telemedicine was seen as an effective pandemic response strategy to allow physicians to manage ambulatory patients with chronic disease while reducing the risks of viral transmission to health care providers and other patients and conserve personal protective equipment (PPE) [2]. The uptake of telemedicine during the first wave of the pandemic was between 38%-77% across different countries with no signs of a return to prepandemic levels [1,3]. With increasing rates of vaccination and a consistent supply of PPE, the long-term sustainability and impact of telemedicine beyond the pandemic is uncertain.

Congestive heart failure (CHF) is an example of an ambulatory sensitive chronic disease where it is presumed that an in-person clinical assessment, including a physical examination, is necessary to provide high-quality care [4]. There have been numerous studies that have demonstrated remote monitoring for patients with CHF, which have led to improved outcomes, including reduced hospitalizations and deaths as an adjunctive strategy; however, to date, no studies have compared telemedicine visits as a substitute to in-person care [5-7]. While telemedicine is generally thought to improve patient experience as it is more convenient with reduced travel time to appointments, there is a worry that telemedicine and the inability to examine the patient physically will lead to increased usage of health services, including more frequent visits, diagnostic testing, and potentially worse clinical outcomes [8-10]. To date, there are limited large-scale studies assessing the impact of telemedicine visits on quality of care on patients with CHF.

The purpose of this study was to investigate the association between telemedicine use and changes in other forms of health care usage and clinical outcomes among patients with CHF from before the COVID-19 pandemic to the early stages of the pandemic, when telemedicine usage became widespread.

## Methods

### Study Design and Data Sources

We conducted a population-based, retrospective cohort study of patients with CHF, using administrative claims data from Ontario, Canada. The following databases were used: (1) Ontario Health Insurance Plan (OHIP), which includes information on all health services delivered by physicians to Ontario patients who are eligible for coverage; (2) the Discharge Abstract Database, which records all inpatient hospital admissions; (3) the National Ambulatory Care Reporting System, which contains data on all hospital- and community-based ambulatory care (including emergency department [ED] visits); (4) Ontario Drug Benefit, which includes data on prescription claims for patients aged >65 years; (5) the Registered Persons Database, which contains demographic information of all patients covered under OHIP; and (6) the CHF database, an Institute for Clinical Evaluative Sciences (ICES) database that uses validated algorithms to identify patients ever diagnosed with CHF, and other ICES-validated disease-specific registries [11]. The Postal Code Conversion File was used to convert all patient postal codes to neighborhood income quintiles. ICES is an independent nonprofit research institute whose legal status under Ontario’s health information privacy law allows it to collect and analyze health care and demographic data without consent for health system evaluation and improvement. Databases were linked using unique encoded identifiers and analyzed at ICES.

### Population

We identified patients diagnosed with heart failure by using a validated algorithm with high sensitivity and specificity [12], who were included if they met all of the following criteria: (1) having a record in the ICES CHF database any time prior to March 14, 2020; (2) having at least one ambulatory visit between March 14 and September 30, 2020; and (3) having at least one hospital admission or ED visit with International Classification of Disease–10th Revision code I50 listed as the most responsible diagnosis in the 3 years prior to their ambulatory visit (Table S1 in Multimedia Appendix 1). We selected March 14, 2020, as the start date of the observation window because it was the day that new temporary billing codes were introduced by the Ontario government, which expanded physician reimbursement of telemedicine services in response to the COVID-19 pandemic [13].

We then stratified the cohort of patients with CHF into 2 groups: a telemedicine group, comprising patients who had at least 2 telemedicine visits, which includes both telephone and video visits, within the observation window (March 14 to September 30, 2020); and an unexposed group, comprising patients who had no more than one telemedicine visit but did have at least one ambulatory visit (in-person or telemedicine) within the observation window. The index visit for each patient was their first telemedicine visit (or first in-person visit for those with zero telemedicine visits during the window). Table S2 in Multimedia Appendix 1 provides the codes used to define telemedicine claims. We excluded patients who were not Ontario residents or had an invalid or missing health card number.

### Propensity Score Matching

To ensure comparability between the telemedicine group and unexposed group, we calculated a propensity score for each patient to represent their probability of receiving telemedicine. Individuals from the telemedicine group and the unexposed group were then matched 1:1 based on their propensity scores using greedy matching algorithms within 0.2 SD. We randomly assigned each individual in the unexposed group an index date to match the distribution of the exposure group index dates. Furthermore, we exact-matched on several key variables: age, sex, and number of hospitalizations owing to CHF in the 3 months prior to the index date. To ensure that matching was successful, the distribution of characteristics in both groups was then compared, and standardized differences greater than 0.1 were considered imbalanced. The following covariates were incorporated into the model that was used to generate individual propensity scores: income quintile, rural residence, number of ED visits owing to heart failure in 12 months prior to the index date, prescription claims for select medication classes in 100 days prior to the index date (angiotensin-converting enzyme inhibitors or angiotensin II receptor blockers, antiplatelets, beta-blockers, aldosterone receptor antagonists, statins, diuretics, nitrates, and digoxin), Charlson comorbidity index in 3 years prior, number of outpatient primary care and cardiology visits in the year prior, diabetes diagnosis any time prior, hypertension diagnosis any time prior, hospitalization for acute myocardial infarction in 3 years prior, peripheral vascular disease within 3 years prior, history of coronary artery disease in 3 years prior, and atrial fibrillation diagnosis in 3 years prior (Table S3 in Multimedia Appendix 1).

### Outcomes

We enumerated the following health care usage outcomes monthly, 12 months before the index date, and over the 90-day period post the index date: number of hospitalizations owing to CHF, hospitalizations owing to cardiovascular disease, all-cause hospitalizations, all-cause ED visits, outpatient primary care visits, repeat outpatient cardiology visits, outpatient cardiology visits with any cardiologist, laboratory claims (ie, hemoglobin A_1c_, lipid profile, complete blood count, and creatinine), cardiac diagnostic tests (transthoracic echocardiogram, cardiac stress test, cardiac catheterization, and Holter monitoring), and new prescription claims.

### Statistical Analysis

We developed a generalized estimating equation (GEE) model for each outcome based on the independent variables time, exposure group, and the time×group interaction. We accounted for correlation due to matching as the GEE could only incorporate one level of clustering. An exchangeable correlation structure was used. Rate ratios, also known as the slope of change over the 15-month period, were calculated for both unexposed and telemedicine groups for each outcome. A rate ratio, or slope, greater than 1 implies that there was a general increase in usage over time for that group. A ratio of the slopes, defined as the slope for the telemedicine group divided by the slope for the unexposed group, was also calculated to compare whether the rate of change over time significantly differed between groups. A ratio of slopes greater than 1 implies that there was higher usage over time in the telemedicine group than in the unexposed group. Absolute rates of usage per 100 person-months over the 15-month period were also calculated for each outcome, along with rate differences to compare between groups. The rate of the unexposed group was subtracted from that of the telemedicine group; therefore, a positive rate difference indicates a higher rate in the telemedicine group. All analyses were performed in SAS (version 9.4; SAS Institute).

### Ethics Approval

Use of these databases for the purposes of this study was authorized under §45 of Ontario’s Personal Health Information Protection Act, which does not require review by a research ethics board. An exemption was also received from the Women’s College Hospital Research Ethics Board (reference number: (REB # 2020-0106-E).

## Results

### Patient Characteristics

Prior to matching, we identified 12,741 eligible patients with CHF in the unexposed group and 33,250 patients with CHF in the telemedicine group (Table 1), and after propensity score matching, 11,131 pairs were identified. Table 1 shows the distribution of baseline patient characteristics in the unexposed versus telemedicine group before and after matching (49% were male; mean age 78.9, SD 12.0 years). Matching successfully balanced characteristics between the 2 groups, as demonstrated by standardized differences of <0.10 for all measured baseline characteristics.

**Table 1:** Baseline characteristics of patients before and after propensity score matching (with standardized differences).

Variables			Before propensity score matching						After propensity score matching				
			Unexposed group (n=12,741)	Telemedicine group (n=33,250)		Standardized difference		Unexposed group (n=11,131)			Telemedicine group (n=11,131)		Standardized difference
**Sex, n (%)**													
	Female	6703 (52.6)		16,111 (48.5)	0.08		5677 (51.0)			5677 (51.0)		0	
	Male	6038 (47.4)		17,139 (51.5)	0.08		5454 (49.0)			5454 (49.0)		0	
Age (years), mean (SD)			79.7 (12.3)	76.9 (11.6)		0.23^a^		78.9 (12.0)			78.9 (12.0)		0
**Charlson comorbidity index, n (%)**													
	0	1469 (11.5)		3828 (11.5)	0		1325 (11.9)			1297 (11.7)		0.01	
	1	2959 (23.2)		7007 (21.1)	0.05		2550 (22.9)			2619 (23.5)		0.01	
	≥2	8313 (65.2)		22,415 (67.4)	0.05		7256 (65.2)			7215 (64.8)		0.01	
Congestive heart failure admission in 3 months prior, n (%)			964 (7.6)	3224 (9.7)		0.08		706 (6.3)			706 (6.3)		0
Congestive heart failure admission in 1 year prior, n (%)			3595 (28.2)	10,513 (31.6)		0.07		3128 (28.1)			2919 (26.2)		0.04
Emergency department visit for congestive heart failure in 1 year prior, n (%)			4228 (33.2)	12,901 (38.8)		0.12^a^		3745 (33.6)			3708 (33.3)		0.01
**Neighborhood income quintile, n (%)**													
	1	3585 (28.1)		8231 (24.8)	0.08		3027 (27.2)			3041 (27.3)		0	
	2	2860 (22.4)		7464 (22.4)	0		2527 (22.7)			2545 (22.9)		0	
	3	2365 (18.6)		6703 (20.2)	0.04		2109 (18.9)			2066 (18.6)		0.01	
	4	2031 (15.9)		5632 (16.9)	0.03		1780 (16.0)			1812 (16.3)		0.01	
	5	1812 (14.2)		5085 (15.3)	0.03		1626 (14.6)			1595 (14.3)		0.01	
**Rurality, n (%)**													
	Rural	1550 (12.2)		2691 (8.1)	0.14^a^		1231 (11.1)			1253 (11.3)		0.01	
	Urban	10,895 (85.5)		30,195 (90.8)	0.16^a^		9696 (87.1)			9682 (87.0)		0	
Prior diabetes, n (%)			6585 (51.7)	19,122 (57.5)		0.12^a^		5941 (53.4)			5863 (52.7)		0.01
Prior hypertension, n (%)			11,620 (91.2)	30,759 (92.5)		0.05		10,188 (91.5)			10,194 (91.6)		0
Acute myocardial infarction admission in 3 years prior, n (%)			954 (7.5)	2551 (7.7)		0.01		859 (7.7)			849 (7.6)		0
Peripheral vascular disease in 3 years prior, n (%)			936 (7.3)	2722 (8.2)		0.03		843 (7.6)			854 (7.7)		0
Coronary artery disease in 3 years prior, n (%)			1694 (13.3)	5366 (16.1)		0.08		1576 (14.2)			1568 (14.1)		0
Atrial fibrillation in 3 years prior			6790 (53.3)	18,330 (55.1)		0.04		5967 (53.6)			5934 (53.3)		0.01
Outpatient primary care visits in 1 year prior, mean (SD)			3.5 (4.5)	5.9 (5.6)		0.47^a^		3.9 (4.6)			3.9 (4.5)		0
Outpatient visits with same cardiologist in 1 year prior, mean (SD)			0.5 (1.2)	1.0 (1.7)		0.34^a^		0.5 (1.2)			0.6 (1.3)		0.02
Outpatient visits with any cardiologist in 1 year prior, mean (SD)			0.9 (1.5)	1.6 (2.0)		0.41^a^		1.0 (1.5)			1.0 (1.5)		0.02
**Prescriptions in 100 days prior, n (%)**													
	Angiotensin-converting enzyme inhibitor or angiotensin II receptor blocker	3703 (29.1)		10,919 (32.8)	0.08		3362 (30.2)			3339 (30.0)		0	
	Antithrombotic	2419 (19.0)		6754 (20.3)	0.03		2119 (19.0)			2179 (19.6)		0.01	
	Beta-blocker	6504 (51.0)		17,944 (54.0)	0.06		5760 (51.7)			5824 (52.3)		0.01	
	Diuretic	5837 (45.8)		15,515 (46.7)	0.02		5137 (46.2)			5180 (46.5)		0.01	
	Calcium channel blocker or statin	7524 (59.1)		21,934 (66.0)	0.14^a^		6855 (61.6)			6951 (62.4)		0.02	
	Nitrate	1142 (9.0)		2687 (8.1)	0.03		967 (8.7)			969 (8.7)		0	
	Aldosterone receptor antagonist	8473 (66.5)		22,416 (67.4)	0.02		7385 (66.3)			7450 (66.9)		0.01	
	Digoxin	1718 (13.5)		4559 (13.7)	0.01		1473 (13.2)			1493 (13.4)		0.01	

^a^Standardized difference>0.1.

### Hospitalizations and ED Visits

**Figure 1:**
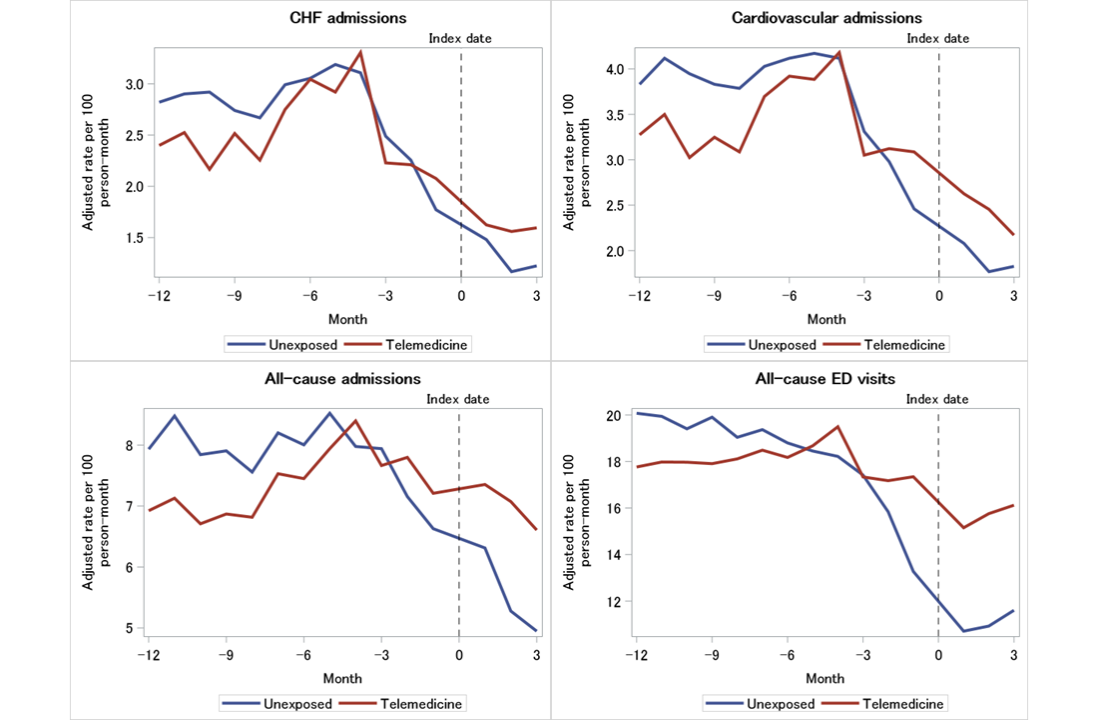
Rate of hospitalizations and emergency department visits by exposure group. CHF: congestive heart failure; ED: emergency department.

Figure 1 illustrates the adjusted rates of hospitalizations and ED visits across time in both the unexposed and telemedicine groups. During the 15-month period starting 12 months before their index visit, which was defined as their first in-person or telemedicine visit during the pandemic, to 3 months post the index date, both groups had a significant reduction in CHF and cardiovascular admissions, though the decrease was greater in the unexposed group. The average monthly decrease in CHF admissions over the 15-month observation period was –5.2% in the unexposed group versus –1.7% in the telemedicine group and –4.7% in the unexposed group versus –2.2% in the telemedicine group for cardiovascular admissions. Similarly, both groups saw declines in monthly all-cause ED visits over the observation period (–3.6% for the unexposed group vs –0.6% for the telemedicine group).

Table 2 reports the rate ratio (slope) and ratio of slope estimates from the GEE model, as well as the absolute rates and accompanying rate differences. The ratio of the slopes indicates a steeper decline in the unexposed group in CHF admissions (ratio of rate ratio [RRR] 1.02, 95% CI 1.02-1.03), cardiovascular admissions (RRR 1.03, 95% CI 1.02-1.04), all-cause admissions (RRR 1.03, 95% CI 1.02-1.04), and any-cause ED visits (RRR 1.03, 95% CI 1.03-1.04). The absolute rate differences were –0.12, –0.15, –0.08, and 0.67 admissions per 100 person-months, respectively.

**Table 2:** Absolute and relative rates by virtual care user group.

Outcomes		Rate ratio or slope^a^ (95% CI)		Ratio of slopes^b^ (95% CI)	Absolute rate per 100 person-month		Rate difference
		Unexposed group	Telemedicine group		Unexposed group	Telemedicine group	
**Hospitalizations and emergency department visits**							
	Congestive heart failure admission	0.95 (0.94-0.96)^c^	0.98 (0.97-0.98)^c^	1.02 (1.02-1.03)^c^	2.47	2.36	–0.12
	Cardiovascular admission	0.95 (0.95-0.96)^c^	0.98 (0.97-0.99)^c^	1.03 (1.02-1.04)^c^	3.39	3.24	–0.15
	Any-cause admission	0.98 (0.97-0.98)^c^	1.00 (1.00-1.01)	1.03 (1.02-1.04)^c^	7.46	7.38	–0.08
	Any-cause emergency department visits	0.96 (0.96-0.96)^c^	0.99 (0.99-0.99)^c^	1.03 (1.03-1.04)^c^	17.17	17.84	0.67
**Physician visits**							
	Primary care visits	0.93 (0.92-0.93)^c^	0.92 (0.92-0.92)^c^	0.99 (0.99-1.00)^c^	28.07	27.49	–0.58
	Visits with the same cardiologist	0.93 (0.92-0.93)^c^	0.93 (0.93-0.94)^c^	1.01 (1.00-1.02)	3.92	4.13	0.22
	Visits with any cardiologist	0.92 (0.92-0.93)^c^	0.93 (0.93-0.94)^c^	1.01 (1.01-1.02)^c^	6.74	7.06	0.32
**Other health care usage**							
	Total laboratory tests	0.97 (0.96-0.97)^c^	0.99 (0.99-0.99)^c^	1.02 (1.02-1.03)^c^	58.48	71.32	12.84
	Total diagnostic tests	0.94 (0.94-0.95)^c^	0.98 (0.98-0.99)^c^	1.04 (1.03-1.05)^c^	10.67	12.10	1.43
	New prescriptions (age>65 years)	0.94 (0.93-0.94)^c^	0.96 (0.95-0.96)^c^	1.02 (1.01-1.03)^c^	22.53	21.59	–0.94

^a^A rate ratio or slope of greater than 1 implies a general increase in health care usage over time, and vice versa.

^b^Ratio of the slopes is defined as the slope for the telemedicine group divided by the slope for the unexposed group. A value greater than 1 implies that there was higher usage over time in the telemedicine group than in the unexposed group.

^c^Statistically significant (95% CI does not include 1, or *P*<.05).

### Physician Visits

**Figure 2:**
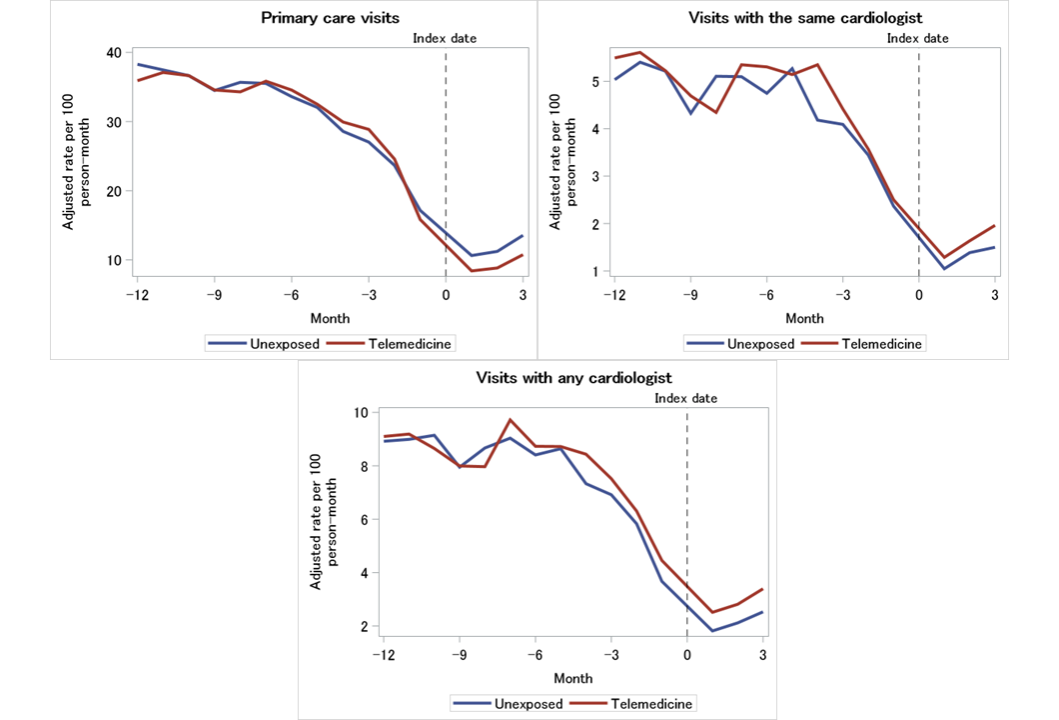
Rate of physician visits by exposure group.

Figure 2 shows the trends in physician visit rates for the unexposed and telemedicine groups. Over the 15-month study period, both groups had a significant monthly decline in primary care visits (–6.1% for the unexposed group vs –6.5% for the telemedicine group), visits with the same cardiologist as the index visit (–5.4% for the unexposed group vs –4.8% for the telemedicine group), and visits with any cardiologist (–6.4% for the unexposed group vs –5.1% in the telemedicine group). When comparing the 2 groups, the decline in the rate of visits with any cardiologist was steeper in the unexposed group than in the telemedicine group (RRR 1.01, 95% CI 1.01-1.02) with an absolute difference of 0.32 visits per 100 person-months; however, the decline in primary care visit rates was steeper in the telemedicine group (RRR 0.99, 95% CI 0.99-1.00) with an absolute difference of –0.58 visits per 100 person-months. There was no significant difference between low and high users in their slopes for visits with the same cardiologist.

### Laboratory Testing, Diagnostic Imaging, and Medication Usage

**Figure 3:**
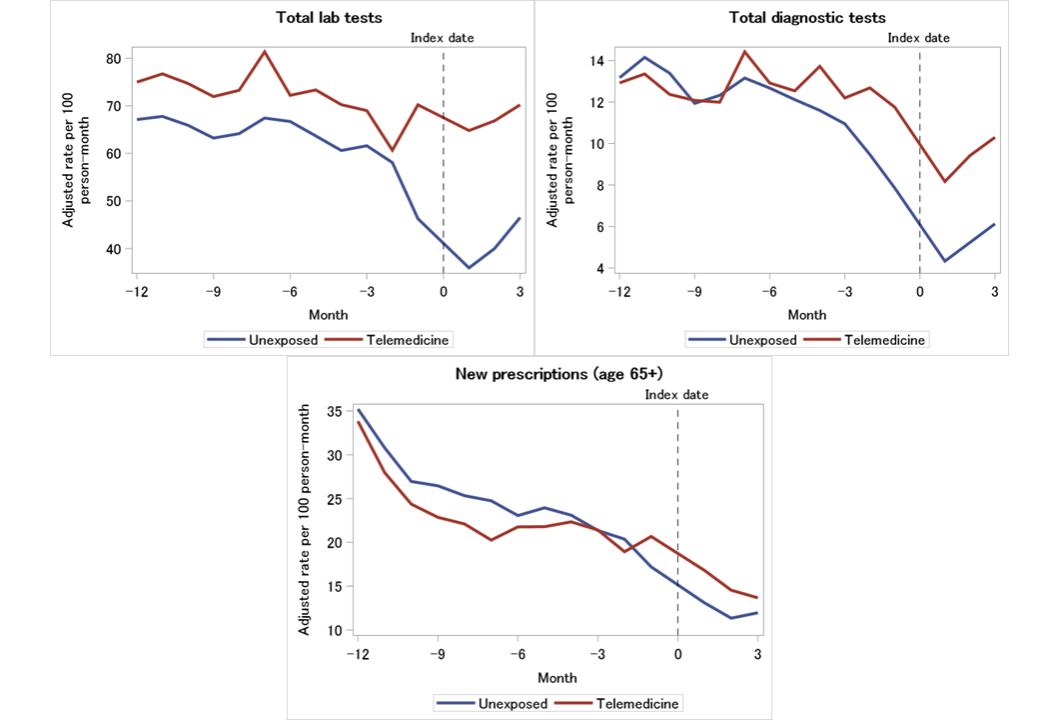
Rate of laboratory tests, diagnostic tests, and prescription claims by exposure group.

Figure 3 displays the monthly ordering rates of laboratory testing, imaging, and medication prescriptions over time. Both the unexposed and telemedicine groups reported a significant decrease across the 15-month observation period in the monthly rates of total laboratory tests (–2.1% for the unexposed group vs –0.2% for the telemedicine group), total diagnostic tests (–3.9% for the unexposed group vs –0.8% for the telemedicine group), and new prescriptions among those aged 65 years and older (–7.1% for the unexposed group vs –5.9% for the telemedicine group). The unexposed group showed a steeper decline in laboratory testing (RRR 1.02, 95% CI 1.02-1.03), diagnostic testing (RRR 1.04, 95% CI 1.03-1.05), and new prescriptions (RRR 1.02, 95% CI 1.01-1.03) than the telemedicine group. The corresponding absolute differences were 12.84, 1.43, and –0.94 tests or claims per 100 person-months, respectively.

## Discussion

### Principal Findings

In this large, population-based study, we aimed to evaluate the impact of telemedicine use on changes in health care usage and outcomes on patients with CHF during the first wave of the COVID-19 pandemic. Both the telemedicine and unexposed groups showed significant reductions in health service use in the months leading up to and during the pandemic. Patients with CHF in the unexposed group saw steeper reductions in hospitalization and ED usage rates than those in the telemedicine group. In addition, patients in the unexposed group had steeper reductions in testing and medication prescriptions. In contrast, the rate of decrease in primary care physician visits was higher in the telemedicine group. To further supplement our findings, we also report difference-in-difference ratios comparing the pre- and postindex rates between exposure groups (Table S4 in Multimedia Appendix 1). These results show that the rate comparisons before and during the pandemic between groups are consistent with our main findings. While the differences we found were significant, the absolute differences between the 2 groups were mostly small, and the clinical significance of these findings are uncertain. However, these results highlight the fact that patients with higher telemedicine usage also seem to have higher usage of many other health care services.

### Comparison to Prior Work

The COVID-19 pandemic led to widespread telemedicine adoption in a very short time frame, with rates of telemedicine usage ranging from 1% before the pandemic to over 70% within weeks of the first wave of the pandemic [3], with over 90% of the visits being facilitated by telephone. Telemedicine was widely seen as a temporary emergency measure designed to quickly provide care to patients with chronic disease while reducing infection risk [2]. Despite initial concerns that telemedicine would compromise the quality of care, our findings demonstrate small, albeit significant differences in hospitalization and ED visit rates, which were generally higher over time within telemedicine compared to in-person care. Prior studies of telemedicine and CHF have reported mixed results, with Klersy et al [14] and Chaudhry et al [15] having failed to demonstrate improvements in CHF outcomes in a large, randomized controlled trial of a telemonitoring solution; however, the more recent Telemedical Interventional Management in Heart Failure II study [5] demonstrated significant reductions in hospitalizations and mortality. These studies, however, were mostly conducted before the pandemic and assessed telemonitoring systems that are adjunctive to physician visits, of which the majority of visits in these studies were conducted in person. This study assessed telemedicine visits as a substitute to in-person physician visits. It is possible that frequent telemedicine visits, which are more easily accessible for frail patients with CHF, may have brought patients to medical attention and facilitated hospitalization. It is also possible that patients who had more frequent telemedicine visits were likely to be acutely decompensating, requiring an ED visit for assessment, particularly when access to in-person care was limited. In contrast to our findings, a few international studies have evaluated telemedicine use in the population of patients with heart failure during the COVID-19 pandemic and found that those accessing telemedicine saw a decrease or no difference in hospitalizations during this time [16,17].

The American College of Cardiology’s CHF guidelines recommend recording volume status and vital signs as part of every clinical assessment [4]. Telemedicine visits limit the ability to conduct a physical examination; hence, some suspected that telemedicine visits would lead to higher use of diagnostic testing in lieu of a clinical examination. Our results suggest higher usage of laboratory and diagnostic testing in the telemedicine group, though the reason for that difference is not easy to ascertain from the data. One possible explanation is that, as stated previously, more diagnostic testing was ordered to augment clinical assessment. Another possible explanation, similar to the explanation around ED visits, is that patients with CHF who were more acute received telemedicine visits and consequently received more diagnostic tests and medication prescriptions. It is interesting that there were only marginal differences in physician visit trends between the 2 groups, however, suggesting that differences in testing and medication ordering were beyond merely increased access to physicians. It is possible that because these patients were more unstable, physicians ordered more testing in advance but only scheduled a visit if the test results indicated an issue for follow-up.

The findings of this study have important implications for the long-term sustainability of telemedicine in a postpandemic era. While telemedicine during the pandemic was mainly used to reduce infection risk and conserve PPE [18], the long-term sustainable PPE supply and readily available COVID-19 vaccines necessitate telemedicine use to align with the quadruple aim of improved patient and provider experience, improved health outcomes, and value for money. Prior studies on telemedicine in CHF seem to demonstrate improved patient satisfaction and potentially improved health outcomes; however, these studies were not population-based [19]. Importantly, CHF telemedicine programs need to integrate fully into the normal delivery of CHF care, including in-person visits, to be effective [18].

### Limitations

The results of this study should be contextualized by some significant limitations. First, although we propensity score–matched high-frequency and low-frequency users or nonusers of telemedicine based on a number of important baseline characteristics, there still exists the potential for unmeasured confounders as administrative data do not account for vital signs, laboratory values, or other markers of disease acuity. Second, these user definitions may not be as applicable as we enter a postpandemic era and away from a “virtual-first” model of care. The study took place within the first wave of the COVID-19 pandemic, when in-person services were being significantly curtailed, which limits the generalizability of the study. Third, we are unable to determine the type of telemedicine platform used—telephone or video—in these encounters, although anecdotal evidence from patients and providers suggests that the majority of visits based in Ontario were conducted over the telephone. Finally, we are also unable to ascertain whether other adjunctive devices, such as wearable devices, were used as part of the telemedicine visit, although those devices were not part of common practice. Despite these limitations, our results provide important observations regarding the use of telemedicine and subsequent health care system usage and patient outcomes.

### Conclusions

In this population-based retrospective cohort study of patients with CHF in Ontario, Canada, we found that telemedicine patients had significantly higher use of health care services over time than low-frequency users or nonusers of telemedicine, although clinically significant differences were minimal for most outcomes. As telemedicine becomes a more widespread and permanent form of care delivery, future research is needed to rigorously assess the optimal use of telemedicine—such as which clinical situations would telemedicine derive the most benefit—and quality of care provided during these interactions in order to determine the sustainability of telemedicine as it is integrated into the health system in a post–COVID-19 era.

## Appendix

Multimedia Appendix 1 Tables S1-S4.

## Abbreviations

CHF: congestive heart failure
CIHI: Canadian Institute for Health Information
ED: emergency department
GEE: generalized estimating equation
ICES: Institute for Clinical Evaluative Sciences
MOH: Ministry of Health
OHIP: Ontario Health Insurance Plan
PPE: personal protective equipment
RRR: ratio of rate ratio

## Data Availability

The data sets generated during or analyzed in this study are not publicly available owing to restricted data sharing agreements with Institute for Clinical Evaluative Sciences (ICES) and the Canadian Institute for Health Information (CIHI), but access to the data may be granted by contacting ICES.
