# Supplementary material for: The Association Between Telemedicine Use and Changes in Health Care Usage and Outcomes in Patients With Congestive Heart Failure: Retrospective Cohort Study
Source: JMIR Cardio. 2022 Aug 4;6(2):e36442. doi: 10.2196/36442 (PMC9359304; doi:10.2196/36442)
Supplement: Multimedia Appendix 1 [file cardio_v6i2e36442_app1.docx]

**Table S1. Inclusion criteria for CHF cohort**

| **Inclusion criteria for CHF cohort** |
| --- |
| Patients were included in the analysis if they met the following criteria:   - Had at least one ambulatory visit between March 14, 2020 and the date that falls 3 months ahead of the max OHIP date (Sep 30, 2020). Ambulatory visit defined as OHIP claim with one of the following locations listed on the claim (‘O’, ‘H’, ‘L’, ‘P’) to define office consultation/ambulatory visits [OHIP].   **AND**   - Patients with heart failure [CHF] diagnosis: Record in ICES CHF database any time prior to March 14^th^, 2020   **AND**   - At least one hospital admission [CIHI-DAD] or emergency department visit [NACRS] claim with HF (ICD-10 code = I50) coded as the most responsible diagnosis (dxtype=’M’ for the DAD and dxtype = main for ED records) but not also as a diagnosis type=’2’ (i.e. post-admit comorbidity) in three years prior to visit with the same IKN |

**Table S2. Telemedicine billing codes**

| **Time Period** | **Billing Codes** | **Modality** |
| --- | --- | --- |
| January 1, 2012- September 30, 2020 | B100, B200, B099 | Video-visit |
| April 1, 2020- September 30, 2020 | B103, B203, B209 | Video-visit |
| March 14, 2020- September 30, 2020 | K080, K081, K082, K083 | All telemedicine modalities |

**Table S3. Variables used in propensity score matching**

|  | **Variable** |
| --- | --- |
| **Hard match** | - Age - Sex - Number of Hospitalizations due to heart failure [DAD, NACRS] in the last 3 months before the index date: claim with ICD-10 code I50 as the most responsible diagnosis and dxtype≠2 (post-admit comorbidity). |
| **Propensity score match** | - Income quintile - Rural residence - Number of ED visits due to heart failure in 12 months before index date [DAD, NACRS]: claim with the ICD-10 code I50. - Prescription claims (dichotomous) in last 100-day prior to index date [ODB]: - ACE-I or ARBs - Antiplatelets - Beta-blockers - Aldosterone receptor antagonists (ARAs) - Statins - Diuretics - Nitrates - Digoxin - Charlson Comorbidity Index (categories) in the 3 years prior to index date. - Physician visits in the year prior to index date (count):   a) Frequency of outpatient primary care visits in past year [OHIP] –  Visits were selected using OHIP var SPEC=00=’general practitioner/family physician’ and we counted the total number of claims with any of the following fee codes: A001, A003, A004, A005, A007, A008, A901, A903, E075, G212, G372, G373, G365, G538, G539, G590, G591, K005, K013, K017, P004, A261, A262, A268, K267, K269, K131, K132.  b) Frequency of repeat outpatient cardiology visits [OHIP]: we counted the total number of claims with any of the following fee codes with the same PHYSNUM on claim as index visit.   - A605 = Consultation - A600 = Comprehensive cardiology consultation - A675 = Limited consultation - A606 = Repeat consultation - A603 = Medical specific assessment - A604 = Medical specific re-assessment - A601 = Complex medical specific re-assessment - A608 = Partial assessment   c) Frequency of outpatient cardiology visits with any cardiologist [OHIP]: we counted the total number of claims with any of the following fee codes with SPEC=60=’Cardiologist’.   - A605 = Consultation - A600 = Comprehensive cardiology consultation - A675 = Limited consultation - A606 = Repeat consultation - A603 = Medical specific assessment - A604 = Medical specific re-assessment - A601 = Complex medical specific re-assessment - A608 = Partial assessment - Diagnosis of Diabetes Y/N [ODD]: Record in the ICES ODD database any time prior to index date - Diagnosis of Hypertension Y/N [HYPER]: Record in the ICES HYPER database any time prior to index date - Acute myocardial infarction hospitalization in 3-year [DAD]: claim with either of the following ICD-10 codes = I21, I22 as the most responsible diagnosis and no dxtype=2 (post-admit comorbidity) - Peripheral vascular disease (PVD) [DAD, CCN] within 3 years prior to index date - ICD-10 codes: I70, I71, I731, I738, I739, I74, I771, I790, I792, K551, K558, K559, Z958, Z959 - Prior history of CAD: patient must meet at least one of the follow criteria within three years prior to their index cardiology visit:  1. Acute MI [DAD, NACRS]: At least one claim with one of the following ICD-10 codes (I21, I22) as the most responsible diagnosis (DAD) or main diagnosis (NACRS) 2. Coronary revascularization (PCI or CABG) [DAD, SDS, CCN]:  - CCI codes for CABG: 1IJ76 - CCI codes for PCI: 1IJ50, 1IJ57GQ, 1IJ54 - CCN definition for PCI: Primary reason for referral is coronary artery disease RemovalReasonCD=’PS’ AND removaldate > 0 AND (Cath_ScheduledPciIND=Y OR Cath_StagedPciIND=Y OR Cath_SSPiIND =Y) - CCN definition for isolated CABG: RemovalReasonCD=’PS’ AND surgery_bypassSurgeryIND=Y and surgery_aorticValvesurgeryIND=N and surgery_mitralvalveSurgeryIND=N and TricuspidvalveSurgeryIND=N and Surgery_otherValveSurgeryIND=N - Diagnosis of Atrial Fibrillation: Any one of the following claims for atrial fibrillation as the primary disease within 3 years prior to the index visit date: - At least 1 DAD record - At least 1 NACRS record - At least 1 rhythm control medication (Amiodarone HCL OR Fecainide Acetate OR Propafenone OR Sotalol HCL) - At least 1 anticoagulant (Warfarin sodium or NOAC) + 1 OHIP claim due to AF - At least 1 cardioversion (OHIP code Z437) + 1 OHIP claim due to AF |

**Table S4. Difference-in-difference (DID) ratios comparing treatment groups pre vs. post index date**

| **Outcome** | **DID ratio (95% CI)^a^** | **P-value** |
| --- | --- | --- |
| CHF admissions | 0.75 (0.65-0.87) | 0.0002* |
| Cardiovascular admissions | 0.72 (0.64-0.81) | <.0001* |
| All-cause admissions | 0.73 (0.68-0.79) | <.0001* |
| All-cause ED visits | 0.69 (0.65-0.73) | <.0001* |
| Primary care visits | 1.25 (1.15-1.36) | <.0001* |
| Visits with same cardiologist | 0.84 (0.72-0.97) | 0.0162* |
| Visits with any cardiologist | 0.76 (0.68-0.85) | <.0001* |
| Total lab tests | 0.69 (0.67-0.72) | <.0001* |
| Total diagnostic tests | 0.60 (0.54-0.65) | <.0001* |
| New prescriptions (age 65+) | 0.75 (0.71-0.80) | <.0001* |

******Statistically significant at p<0.05*^a^ Interpretation: DID ratio is calculated as follows: $\frac{pre-index rate\div post-index rate \left( telemedicine group \right)}{pre-index rate\div post-index rate (unexposed group)}$

A DID ratio of less than 1 indicates that there was a steeper decline in rate from pre- to post-index date in the unexposed group (vs. telemedicine group). A DID ratio of greater than 1 indicates that there was a steeper decline in rate from pre- to post-index date in the telemedicine group (vs unexposed group).
